# Uncovering a novel molecular mechanism for scavenging sialic acids in bacteria

**DOI:** 10.1074/jbc.RA120.014454

**Published:** 2020-07-15

**Authors:** Andrew Bell, Emmanuele Severi, Micah Lee, Serena Monaco, Dimitrios Latousakis, Jesus Angulo, Gavin H. Thomas, James H. Naismith, Nathalie Juge

**Affiliations:** 1 Gut Microbes and Health Institute Strategic Programme, Quadram Institute Bioscience, Norwich, United Kingdom; 2 Department of Biology, University of York, York, United Kingdom; 3 Division of Structural Biology, University of Oxford, Headington, Oxford, United Kingdom; 4 School of Pharmacy, University of East Anglia, Norwich Research Park, Norwich, United Kingdom; 5 Departamento de Química Orgánica, Universidad de Sevilla, Sevilla, Spain; 6 Instituto de Investigaciones Químicas (CSIC-US), Sevilla, Spain

**Keywords:** sialic acid, oxidoreductase, 2,7-anhydro-Neu5Ac, STD NMR, gut microbiota, mucin glycosylation, Ruminococcus gnavus, Escherichia coli, sialic acid transporters, symbiosis, sialic acid, microbiology, nuclear magnetic resonance (NMR), Escherichia coli (E. coli), oxidation-reduction (redox), 2,7-anhydro-Neu5AC, gut symbiosis

## Abstract

The human gut symbiont *Ruminococcus gnavus* scavenges host-derived *N*-acetylneuraminic acid (Neu5Ac) from mucins by converting it to 2,7-anhydro-Neu5Ac. We previously showed that 2,7-anhydro-Neu5Ac is transported into *R. gnavus* ATCC 29149 before being converted back to Neu5Ac for further metabolic processing. However, the molecular mechanism leading to the conversion of 2,7-anhydro-Neu5Ac to Neu5Ac remained elusive. Using 1D and 2D NMR, we elucidated the multistep enzymatic mechanism of the oxidoreductase (*Rg*NanOx) that leads to the reversible conversion of 2,7-anhydro-Neu5Ac to Neu5Ac through formation of a 4-keto-2-deoxy-2,3-dehydro-*N*-acetylneuraminic acid intermediate and NAD^+^ regeneration. The crystal structure of *Rg*NanOx in complex with the NAD^+^ cofactor showed a protein dimer with a Rossman fold. Guided by the *Rg*NanOx structure, we identified catalytic residues by site-directed mutagenesis. Bioinformatics analyses revealed the presence of *Rg*NanOx homologues across Gram-negative and Gram-positive bacterial species and co-occurrence with sialic acid transporters. We showed by electrospray ionization spray MS that the *Escherichia coli* homologue YjhC displayed activity against 2,7-anhydro-Neu5Ac and that *E. coli* could catabolize 2,7-anhydro-Neu5Ac. Differential scanning fluorimetry analyses confirmed the binding of YjhC to the substrates 2,7-anhydro-Neu5Ac and Neu5Ac, as well as to co-factors NAD and NADH. Finally, using *E. coli* mutants and complementation growth assays, we demonstrated that 2,7-anhydro-Neu5Ac catabolism in *E. coli* depended on YjhC and on the predicted sialic acid transporter YjhB. These results revealed the molecular mechanisms of 2,7-anhydro-Neu5Ac catabolism across bacterial species and a novel sialic acid transport and catabolism pathway in *E. coli*.

The sialic acids comprise a family of 9-carbon sugar acids found predominantly on cell-surface glycans of humans and other animals (1). Sialic acids are subject to a remarkable number of modifications, generating more than 50 structurally distinct molecules. Their terminal location makes them a preferential target for interaction with viruses and microorganisms at mucosa surfaces across the body (1). *N*-Acetylneuraminic acid (Neu5Ac), the most common form of sialic acid in humans, is a major epitope of mucin glycans that can serve as a metabolic substrate to the gut bacteria that have adapted to the mucosal environment (2). Most of our knowledge on sialic acid metabolism in bacteria comes from the study of Neu5Ac using the model organism *Escherichia coli* (3). We previously reported that sialic acid metabolism is vital to the ability of *Ruminococcus gnavus* strains to utilize mucin as a nutrient source through the production of 2,7-anhydro-Neu5Ac derivative (4, 5).

*R. gnavus* is a human gut symbiont that plays a major role in human health and disease. *R. gnavus* is widely distributed among individuals being represented in the most common 57 species present in ≥90% of individuals (6). Colonization by *R. gnavus* has been found in infants during the first days of life. *R. gnavus* is in the top 15 species showing abundance in both adult and infant gut-enriched genes, supporting *R. gnavus* adaptation to the intestinal habitat throughout life (6, 7). Further, *R. gnavus* has been associated with an increasing number of intestinal or extraintestinal diseases, including inflammatory bowel disease (8–16).

The mucin-foraging strategy of *R. gnavus* is strain-specific (5) and associated with the expression of an intramolecular *trans*-sialidase (IT-sialidase) that targets and cleaves off terminal α2-3–linked Neu5Ac from glycoproteins, releasing 2,7-anhydro-Neu5Ac instead of Neu5Ac (4, 17, 18). We unraveled the molecular pathway leading to the transport and metabolism of 2,7-anhydro-Neu5Ac in *R. gnavus* ATCC 29149 (19). The 2,7-anhydro-Neu5Ac compound binds specifically to the substrate-binding protein (*Rg*SBP), which forms part of an ABC sialic acid transporter in *R. gnavus*. Once inside the cell, 2,7-anhydro-Neu5Ac is converted into Neu5Ac via a novel enzymatic reaction catalyzed by an oxidoreductase, *Rg*NanOx. Following this conversion, Neu5Ac is then catabolized into *N*-acetylmannosamine (ManNAc) and pyruvate via the action of a Neu5Ac-specific aldolase (19). We confirmed the importance of this metabolic pathway *in vivo* by generating a *R. gnavus nan* cluster deletion mutant that lost the ability to grow on sialylated substrates. We showed that in gnotobiotic mice colonized with *R. gnavus* WT and mutant strains, the fitness of the *nan* mutant was significantly impaired as compared with the WT strain with a reduced ability to colonize the mucus layer (19).

The novel oxidoreductase identified in our work showed to catalyze the conversion 2,7-anhydro-Neu5Ac to Neu5Ac in the presence of NAD^+^ in a reversible manner (19). Recently, the crystal structure of the *Rg*NanOx homologue in *E. coli*, YjhC, was solved in complex with NAD^+^ (20). However, the mechanism of action of these newly identified enzymatic activities remained undefined. Here, using a combination of *in silico*, molecular, biochemical, and structural approaches, we elucidated the molecular mechanism of *Rg*NanOx and showed that homologous enzymes are present across both Gram-positive and Gram-negative bacteria and are associated with different classes of predicted transporters. We validated these data *in vitro* and further unraveled the 2,7-anhydro-Neu5Ac catabolism in *E. coli*.

## Results

### Characterization of R. gnavus oxidoreductase (RgNanOx) reaction revealed a novel mechanism involving a 4-keto-2-deoxy-2,3-dehydro-N-acetylneuraminic acid (4-keto-DANA) intermediate

*Rg*NanOx (RUMGNA_02695) from *R. gnavus* ATCC 29149 catalyzes the equilibration of 2,7-anhydro-Neu5Ac and Neu5Ac (19). To gain insights into the mechanism of action of the enzyme, the conversion of 2,7-anhydro-Neu5Ac to Neu5Ac was monitored by ^1^H NMR for 24 h. Comparison of samples differing only in the solvent (light water (H_2_O) *versus* deuterated water (D_2_O) at the same reaction time points showed the loss of specific ^1^H signals from protons attached at C3 and C5 in D_2_O, indicating solvent exchange. The loss of these signals led to simplification in the splitting of the neighboring proton signals (Fig. S1).

**Figure 1.**
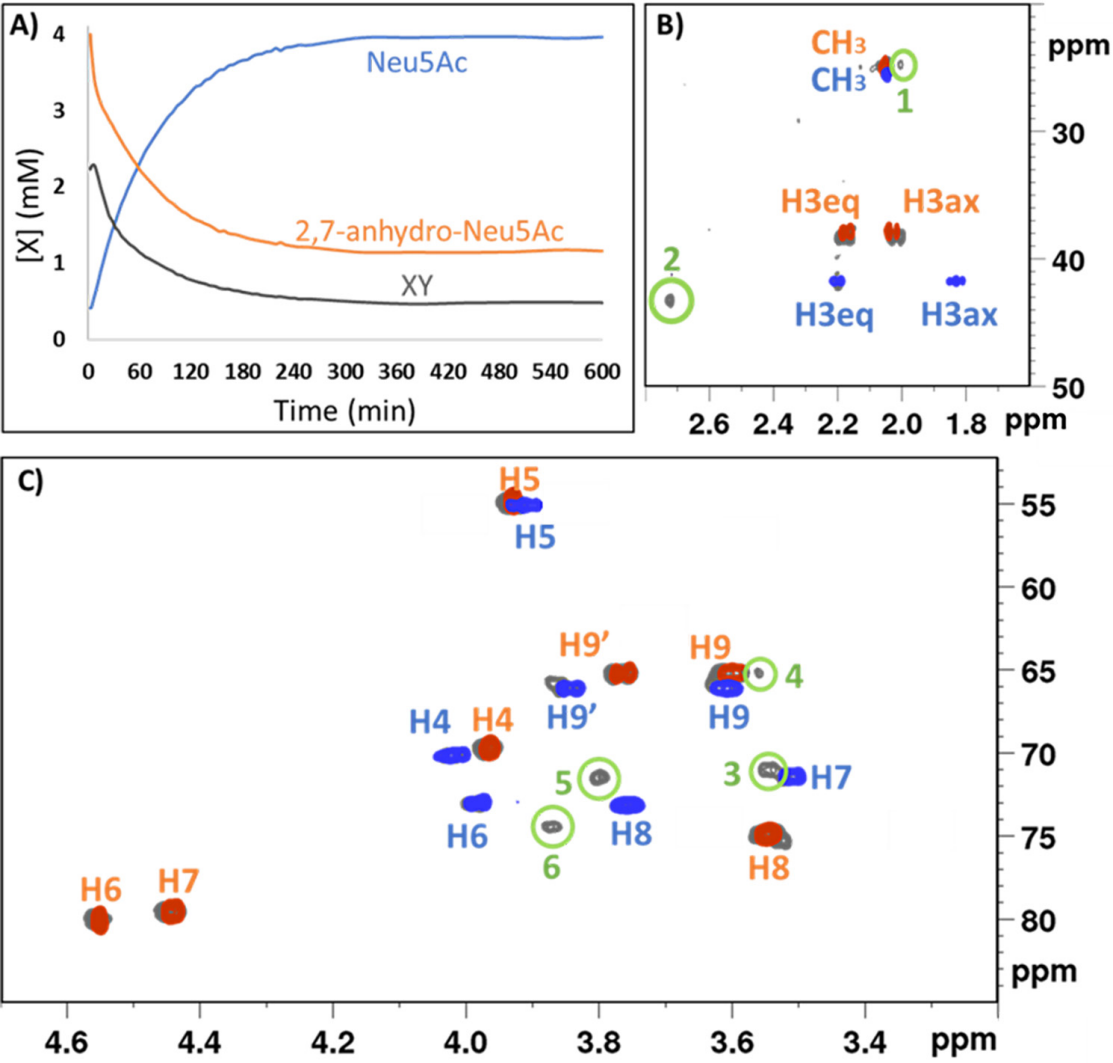
**NMR characterization of the *Rg*NanOx-catalyzed enzymatic reaction.** *A*, ^1^H 1D NMR analysis of the conversion of 2,7-anhydro-Neu5Ac to Neu5Ac via the XY intermediate; evolution of the concentrations of substrate, product, and intermediate of the reaction, [X] (mm), by integration of the acetyl methyl groups for Neu5Ac (*blue*), 2,7-anhydro-Neu5Ac (*orange*), or XY intermediate (*gray*). *B* and *C*, superposition of the ^1^H,^13^C HSQC reference spectra of Neu5Ac (*blue*) and 2,7-anhydro-Neu5Ac (*orange*) and ^1^H,^13^C HSQC reaction mixture at 30 min (where the XY peak is observed to peak, *gray*). Proton-carbon peaks corresponding to neither Neu5Ac nor 2,7-anhydro-Neu5Ac are *numbered* from *1* to *6* and *circled* in *green*.

Analysis of the reaction curve, obtained by monitoring the signals from the methyl protons of the acetamide group at C5, revealed the presence of a new (third) molecule (XY). This molecule is formed with a very fast kinetics, reaching its highest concentration at very early time (Fig. 1*A*). The reaction mixture was analyzed by 2D NMR during the interval at which the intermediate was at its highest concentration, using a 600-MHz spectrometer equipped with a cryoprobe. The comparison of the 2D ^1^H,^13^C HSQC spectra of the substrate (2,7-anhydro-Neu5Ac), the product (Neu5Ac), and the reaction mixture at 30 min allowed the identification of an additional set of cross-peaks that did not belong to the substrate or to the product and were therefore assigned to the intermediate (Fig. 1, *B* and *C*). Specifically, the presence of a heteronuclear cross-peak at 2.73 ppm/43.1 ppm (^1^H/^13^C) (signal 2 in Fig. 1*B*), characteristic of a proton in α to a keto group, strongly suggested the intermediate to be a keto-sugar. Homonuclear 2D ^1^H,^1^H COSY and ^1^H,^1^H TOCSY experiments showed connectivity of this proton to one and two other protons, respectively (Fig. S2). Signal 2, at 2.73 ppm, corresponds then to a proton neighboring a carbonyl on one side and at least two other protons on the other side with chemical shifts in the 3–4 ppm region. Analysis of the 3–4 ppm spectral region, where standard sugar-ring C-H signals typically show, suggests that the intermediate chemical shifts are closer to Neu5Ac than to 2,7-anhydro-Neu5Ac (Fig. S3). Based on this information, we proposed that in this species, the glycerol moiety is in the open form, indicating that the 2,7-anhydro bond is broken in the intermediate. The formation of such an oxidized intermediate (*i.e.* the keto-sugar), as detected by NMR, is compatible with the regeneration of NAD^+^ observed previously (19). As the final result of the reaction is a reductive opening of the ring through the O7–C2 bond, a preliminary oxidation must occur earlier in the mechanism.

**Figure 2.**
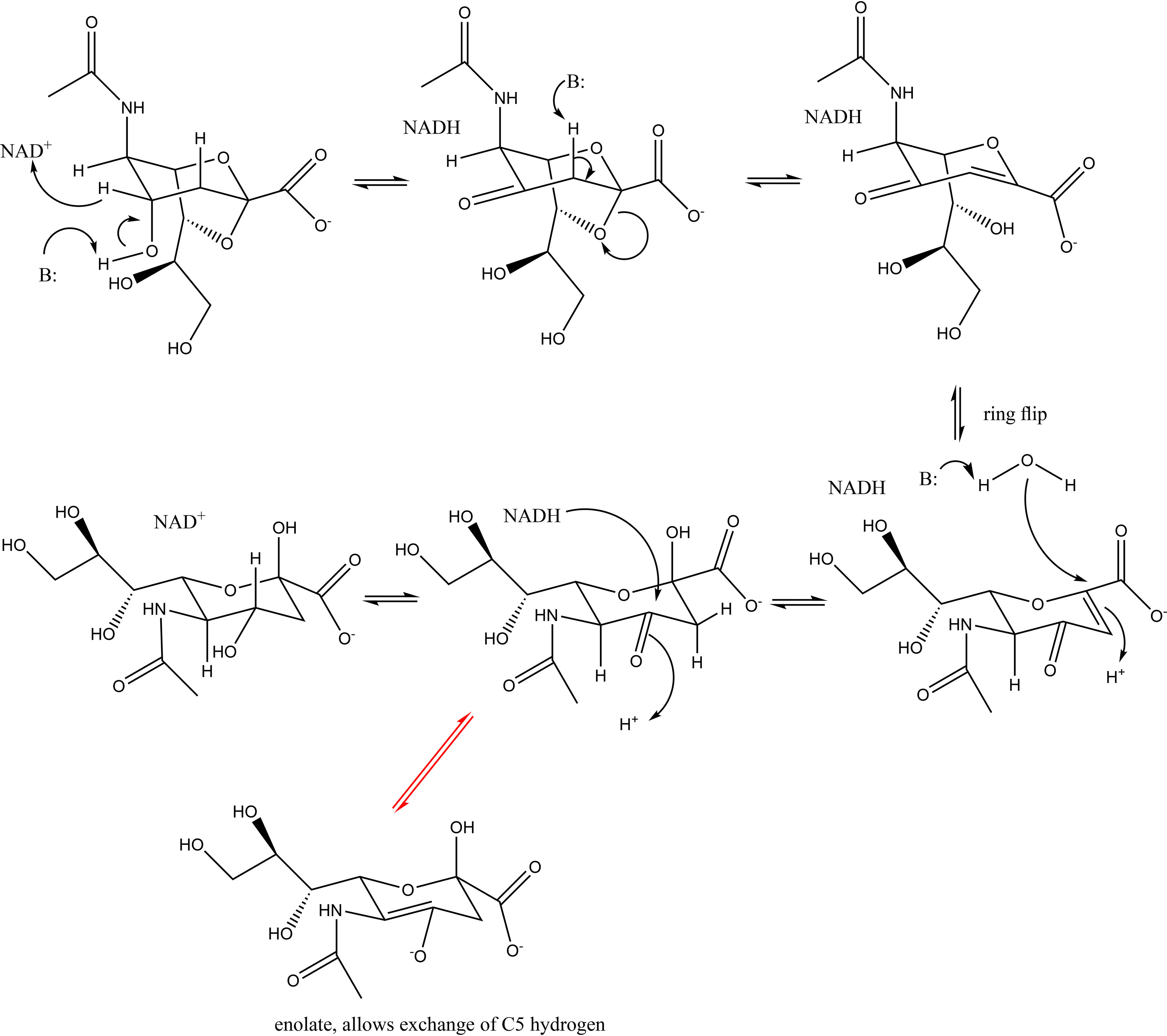
**Proposed mechanism for the reversible conversion of Neu5Ac to 2,7-anhydro-Neu5Ac by *Rg*NanOx.** The reaction is shown in the favorable direction converting 2,7-anhydro-Neu5Ac (1) to Neu5Ac (6). The order of events taking compound 2 to compound 4, including the opening of the 2,7 secondary ring and the primary ring flip, has yet to be determined. The *red arrows* indicate the keto enol tautomerization of compound 5 that allows for the C5 hydrogen exchange.

**Figure 3.**
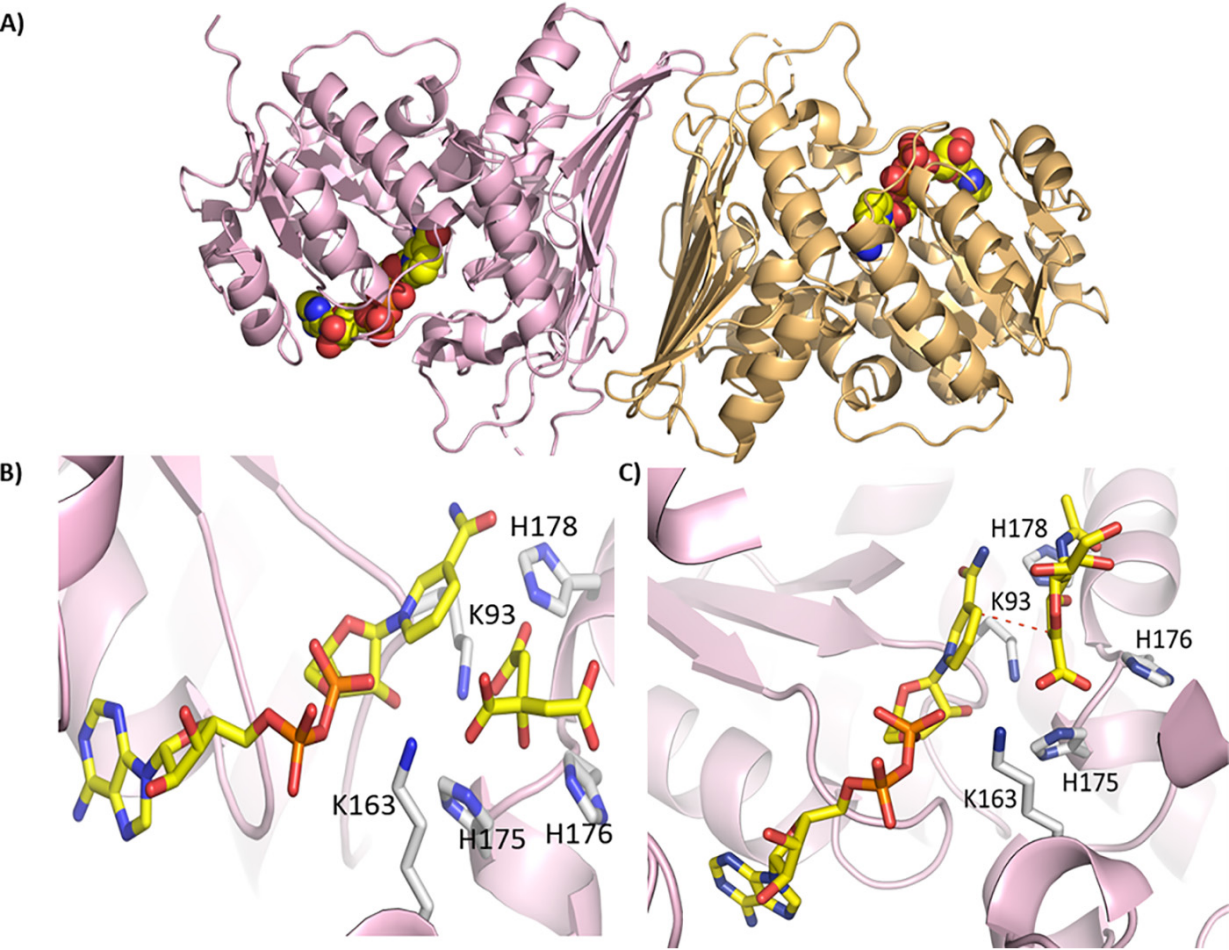
**Crystal structure of *Rg*NanOx.** *A*, dimeric structure of *Rg*NanOx shown in *cartoon format* with the NAD cofactor bound (*spheres*). *B*, structure of putative active site of *Rg*NanOx; the protein backbone is shown in *cartoon* with residues NAD and citric acid shown in *sticks*. *C*, crystal structure of *Rg*NanOx with key residues marked and DANA modeled into the active site. The *red line* marks the trajectory of hydride transfer.

Based on this analysis, we propose a multistep mechanism for the reversible conversion of 2,7-anhydro-Neu5Ac to Neu5Ac by *Rg*NanOx (Fig. 2). In the first step, 2,7-anhydro-Neu5Ac is oxidized at the C4 keto by NAD^+^ cofactor. The proton at C3 is now α to the keto group and thus more acidic and can be abstracted in an anti-periplanar elimination reaction in which the 2,7-anhydro bond is also broken. The resulting compound, 4-keto-DANA, is consistent with 2D NMR, and the conjugation would be expected to stabilize the intermediate (full NMR assignment of 4-keto-DANA is given in Table S1). 4-Keto-DANA exists as an equilibrium between two ring-flipped forms; however, the form with the equatorial glycerol and *N*-acetyl substituents would be expected to predominate. In this ring-flipped form, a Michael addition of water to C2 hydrates the C2=C3 double bond with a proton from the solvent added to C3. The addition reaction would be expected to follow the expected anti-periplanar geometry. This is consistent with the NMR observation that the axial, not equatorial proton, undergoes exchange (Fig. S1). The product of the addition (2) is a 4-keto-Neu5Ac, in which the proton at C5 is now α to the keto and acidic. This acidic proton will exchange with solvent by the well-known keto enol tautomerization reaction, consistent with the NMR data (Fig. S1). The 4-keto-Neu5Ac (2) is finally reduced by NADH to yield the final Neu5Ac product and NAD^+^. The regeneration of NAD^+^ also explains why no net change in the ratio of NAD^+^ and NADH was observed in the enzymatic reaction (19).

### The crystal structure of RgNanOx in complex with NAD^+^ confirms structural homology with short-chain dehydrogenase/reductases

The crystal structure of the recombinant *Rg*NanOx in complex with the NAD^+^ cofactor was solved at 2.58 Å and subsequently at 1.74 Å using molecular replacement with an oxidoreductase from *Agrobacterium radiobacter* as a model (Protein Data Bank (PDB) entry 5UI9). The protein shows a Rossman fold typical of NAD-binding protein of the Gfo/Idh/MocA class (21) characterized by a central β-sheet with helices on either side. *Rg*NanOx forms a dimer in the asymmetric unit, and the NAD^+^ cofactor can be seen bound to the protein (Fig. 3*A*). A structural similarity search (22) identifies multiple oxidoreductase enzymes, all of which share the same Rossman fold and location of the nucleotide-binding site. Oxidoreductase proteins typically have a catalytic triad of K (found in the EKP motif), D, and H (often found as a D*XXX*H motif; in some enzymes Y replaces the H) and a fourth residue, which is positively charged (21). *Rg*NanOx has Lys-93 and His-178 which correspond to the Lys and His of the catalytic triad, and Lys-163 occupies the “fourth” position (23). However, *Rg*NanOx has His-175, which occupies the position typical for the Asp in the catalytic triad (23). The closest structural match (0.8 Å over 341 residues) is the recently solved crystal structure of YjhC oxidoreductase from *E. coli* (Fig. 3*B*) (20), which also has a H*XX*H motif. In the *Rg*NanOx structure, there is additional density adjacent to the nicotinamide ring that, given the high resolution of the second structure, we were able to unambiguously identify as citric acid from the crystallization buffer.

**Figure 4.**
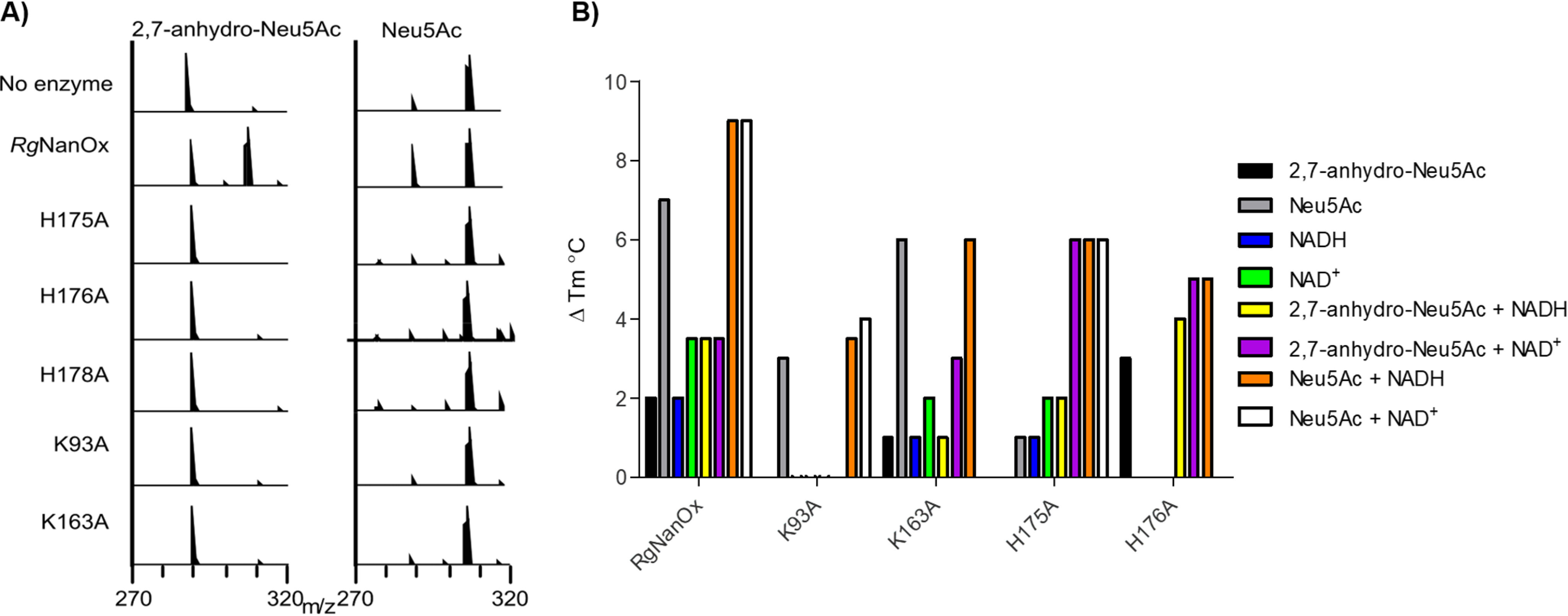
**Analysis of *Rg*NanOx mutants.** *A*, ESI-MS analysis of the enzymatic reaction between *Rg*NanOx mutants and 2,7-anhydro-Neu5Ac (290; *left*) or Neu5Ac (308; *left*). *B*, DSF analysis of *Rg*NanOx mutants binding to NAD/H cofactor and sialic acid substrates. The Δ*T_m_* is shown compared with the *T_m_* of the protein alone.

Using the high-resolution structure, we used a simple modeling approach to place a molecule of DANA a transition state analog inhibitor of sialidases, in *Rg*NanOx active site by overlapping the carboxylate acid of the DANA with each of the three carboxylate groups of citric acid. Next we manually positioned the sugar such that the H3 of the atom ring pointed toward the C4′ of the nicotinamide, as would be required for hydride transfer. These models were then minimized, and we investigated whether the H4 atom of DANA was still able to transfer to nicotinamide. Only one position (Fig. 3*C*) where the DANA carboxylate was placed on the 2-carboxylic acid of citric remained positioned for hydride transfer. The goal of modeling was not to generate a precise model of the substrate protein interactions but rather, along with sequence comparisons, generate hypotheses for site-directed active-site mutagenesis. The model indicated that His-178, predicted to be a catalytic residue, is positioned to remove the proton from O4 during the oxidation step. His-176 is plausibly positioned to undertake proton transfer with the O7 of the substrate glycerol that the mechanism requires. His-175 interacts with the negatively charged carboxylic acid in the model but could play a role in proton transfer at the substrate C3 atom. Lys-93 binds to NAD^+^, is part of the catalytic constellation, and is also within a plausible distance for proton transfer to C3. Lys-163 binds to the NAD^+^ co-factor and interacts with the substrate carboxylate. The following mutants of *Rg*NanOx were constructed: K93A, K163A, H175A, H176A, and H178A. The purified mutants lost enzymatic activity, as demonstrated by electrospray ionization spray MS (ESI-MS) (Fig. 4*A*), supporting the hypothesis that they are catalytically important. K93A lost the ability to bind NAD^+^; as did H176A, to some extent. The other mutants appeared able to retain substrate binding (Fig. 4*B*).

### Catabolism of 2,7-anhydro-Neu5Ac by E. coli requires a RgNanOx homologue (YjhC) and a novel transporter

**Figure 5.**
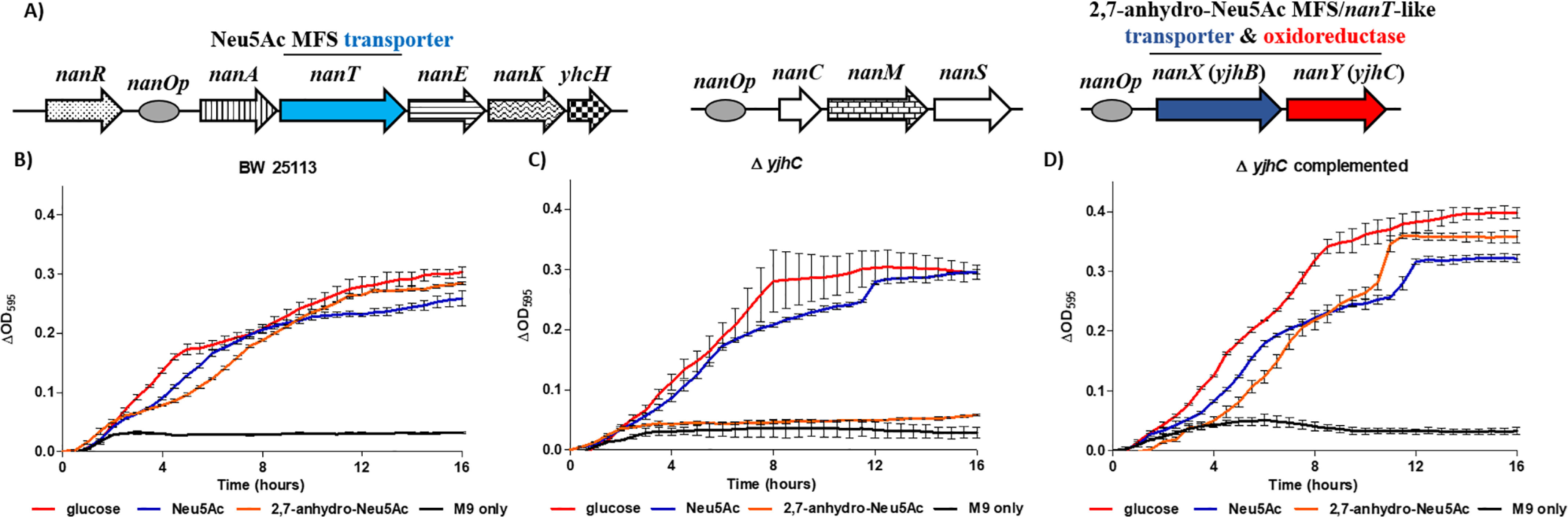
**Structure of the sialometabolic *nan* regulon of *E. coli* K12 strains and the role of YjhC in sialometabolism by *E. coli* BW25113.** *A*, structure of the complete sialometabolic regulon of *E. coli* K12 strains. The repressor NanR controls expression of only these three loci in *E. coli* (25), with *nanOp* indicating the position of NanR-regulated promoters. The first locus is the core *nanATEKyhcH* operon for Neu5Ac uptake and dissimilation into the cytoplasm (62). The two “accessory” loci contain the *nanCMS* operon, for Neu5Ac uptake through the outer membrane, sialic acid mutarotation, and processing of *O*-acetylated sialic acids in periplasm (63–65), and the *nanXY* (*yjhBC*) operon here characterized as being required for 2,7-anhydro-Neu5Ac uptake and utilization. *B–D*, growth of *E. coli* on different carbon sources. All strains were grown on 2,7-anhydro-Neu5Ac (*orange*), Neu5Ac (*blue*), glucose (*red*), or M9 medium alone (*black*) in 200-µl microtiter plates. ΔOD_595_ for triplicate experiments is shown: BW25113 (*B*), Δ*yjhC* (*C*), and complemented *yjhC* (*D*). *Error bars*, S.E. of three biological repeats.

**Figure 6.**
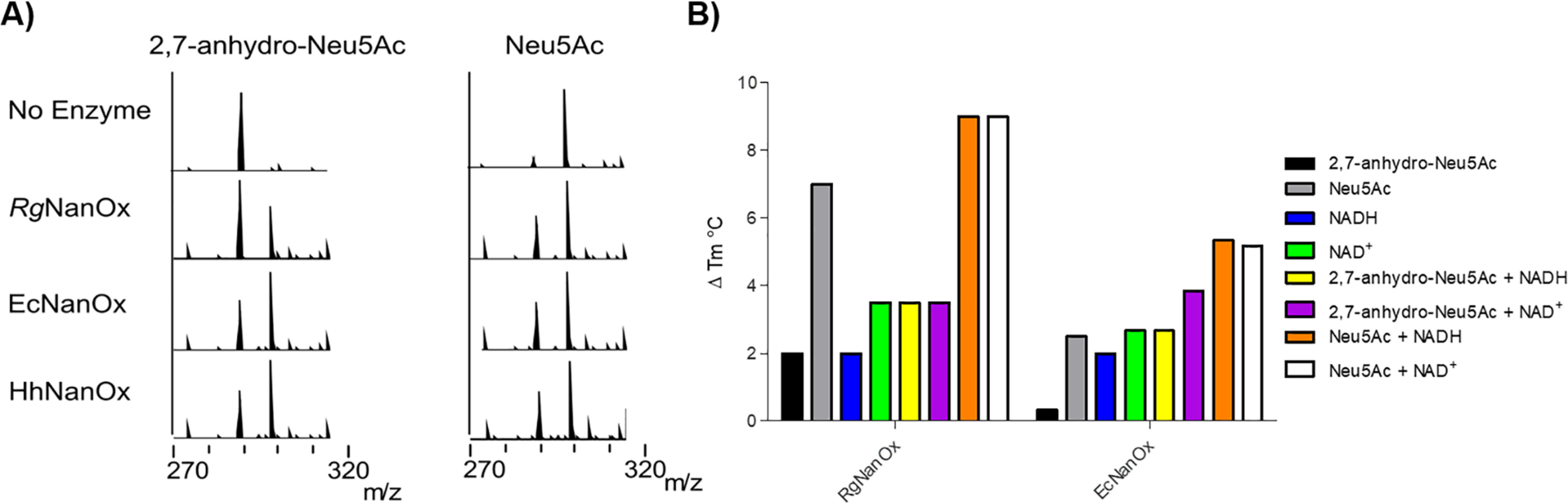
**Characterization of *Ec*NanOx and *Hh*NanOx.** *A*, ESI-MS analysis of the enzymatic reaction of *Rg*NanOx, *Ec*NanOx, and *Hh*NanOx with 2,7-anhydro-Neu5Ac (*left*) or Neu5Ac (*right*). *B*, DSF analysis of *Rg*NanOx and *Ec*NanOx, the Δ*T_m_* is shown compared with the *T_m_* of the protein alone.

Sequence similarity network analysis of the *R. gnavus* Nan cluster (responsible for 2,7-anhydro-Neu5Ac metabolism) identified the presence of *Rg*NanOx homologues in a number of organisms (19). One such example was the model Gram-negative human commensal *E. coli* K-12, the organism in which the genes for Neu5Ac catabolism were first discovered (24, 25). In *E. coli*, the homologue of *Rg*NanOx is part of a two-gene operon, *yjhBC*, which is one of only three operons in *E. coli* regulated by the transcription factor NanR as reported previously (25) (Fig. 5*A*). Here, we demonstrated that *E. coli* could grow on 2,7-anhydro-Neu5Ac as a sole carbon source (Fig. 5*B*), reaching growth yields similar to that obtained when *E. coli* was grown on Neu5Ac. Deletion of *yjhC* resulted in loss of growth on 2,7-anhydro-Neu5Ac but not on Neu5Ac (Fig. 5*C*), which could be complemented in *trans* with *yjhC* (Fig. 5*D*), suggesting that the gene encodes an equivalent protein to *Rg*NanOx. To test this hypothesis, the YjhC protein was recombinantly expressed and purified, and its activity against 2,7-anhydro-Neu5Ac and Neu5Ac was analyzed by ESI-MS. The purified enzyme was shown to be active against both substrates, reaching an equilibrium of 2:1 Neu5Ac/2,7-anhydro-Neu5Ac, in line with that of *Rg*NanOx (19) (Fig. 6*A*). Differential scanning fluorimetry (DSF) analyses confirmed the binding of YjhC to the substrates 2,7-anhydro-Neu5Ac and Neu5Ac as well as to co-factor NAD or NADH (Fig. 6*B*), as shown previously for *Rg*NanOx (19). The knowledge of the biochemical and physiological function of YjhC is particularly useful, given that the 3D structure of this protein has also recently been solved (20).

**Figure 7.**
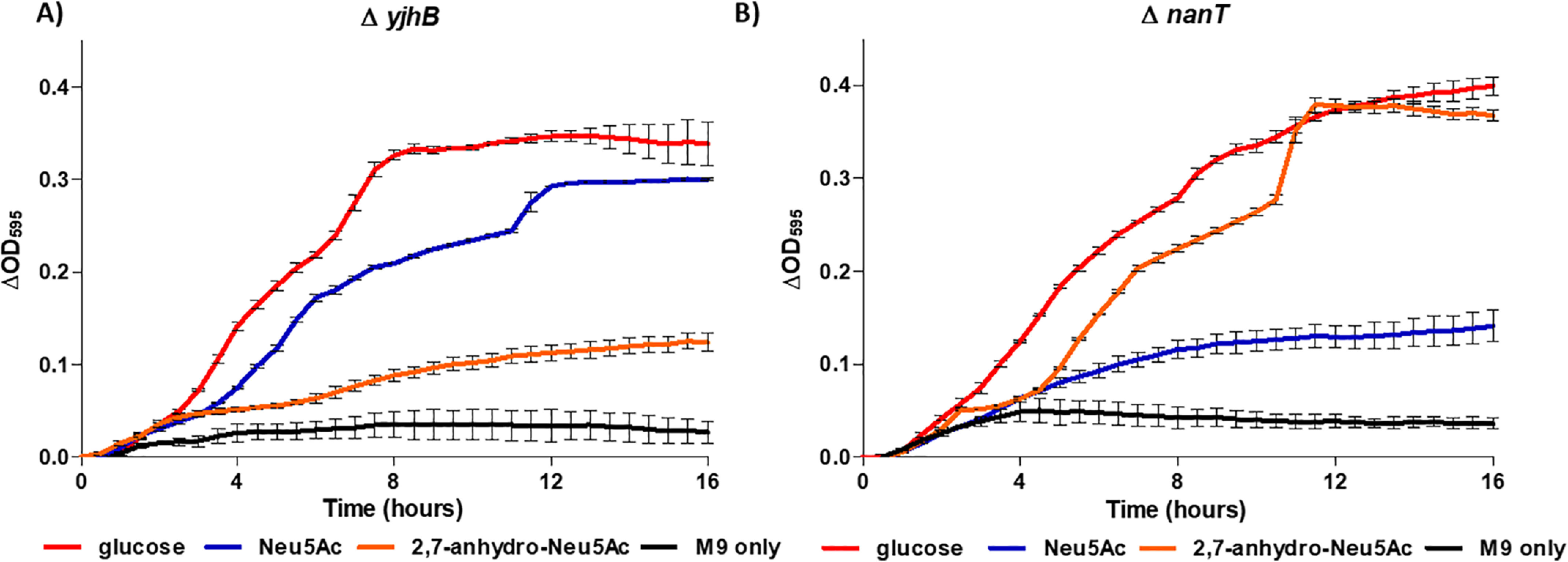
**Growth of sialometabolism *E. coli* BW25113 transporter mutants.** Both mutants were grown on 2,7-anhydro-Neu5Ac (*orange*), Neu5Ac (*blue*), glucose (*red*), or M9 medium alone (*black*) in 200-µl microtiter plates. ΔOD_595_ for triplicate experiments is shown: Δ*nanT* (*A*) and Δ*yjhB* (*B*). *Error bars*, S.E. of three biological repeats.

The first gene in the *yjhBC* operon, *yjhB*, encodes a major facilitator superfamily (MFS) transporter protein that shows homology (35% identify, 55% similarity) to NanT, the known Neu5Ac transporter in *E. coli* (24, 26, 27). Deletion of *nanT* leads to a complete loss of growth on Neu5Ac, suggesting that YjhB cannot transport this particular sialic acid (28) (Fig. 7*A*). Similar to the phenotype observed with the Δ*yjhC* strain, the Δ*yjhB* mutant was also unable to grow on 2,7-anhydro-Neu5Ac but could grow on Neu5Ac (Fig. 7*B*). The co-expression of these two genes and the requirement of YjhB for growth on 2,7-anhydro-Neu5Ac suggest that YjhB is a novel MFS transporter for 2,7-anhydro-Neu5Ac and that these two genes together form an “accessory” operon to allow *E. coli* to scavenge a wider range of sialic acids that are available in the human gut. We propose to rename these genes *nanXY*, because the function of the final NanR-regulated operon has been elucidated through this work.

### Catabolism of 2,7-anhydro-Neu5Ac is widespread across the bacterial kingdom

**Figure 8.**
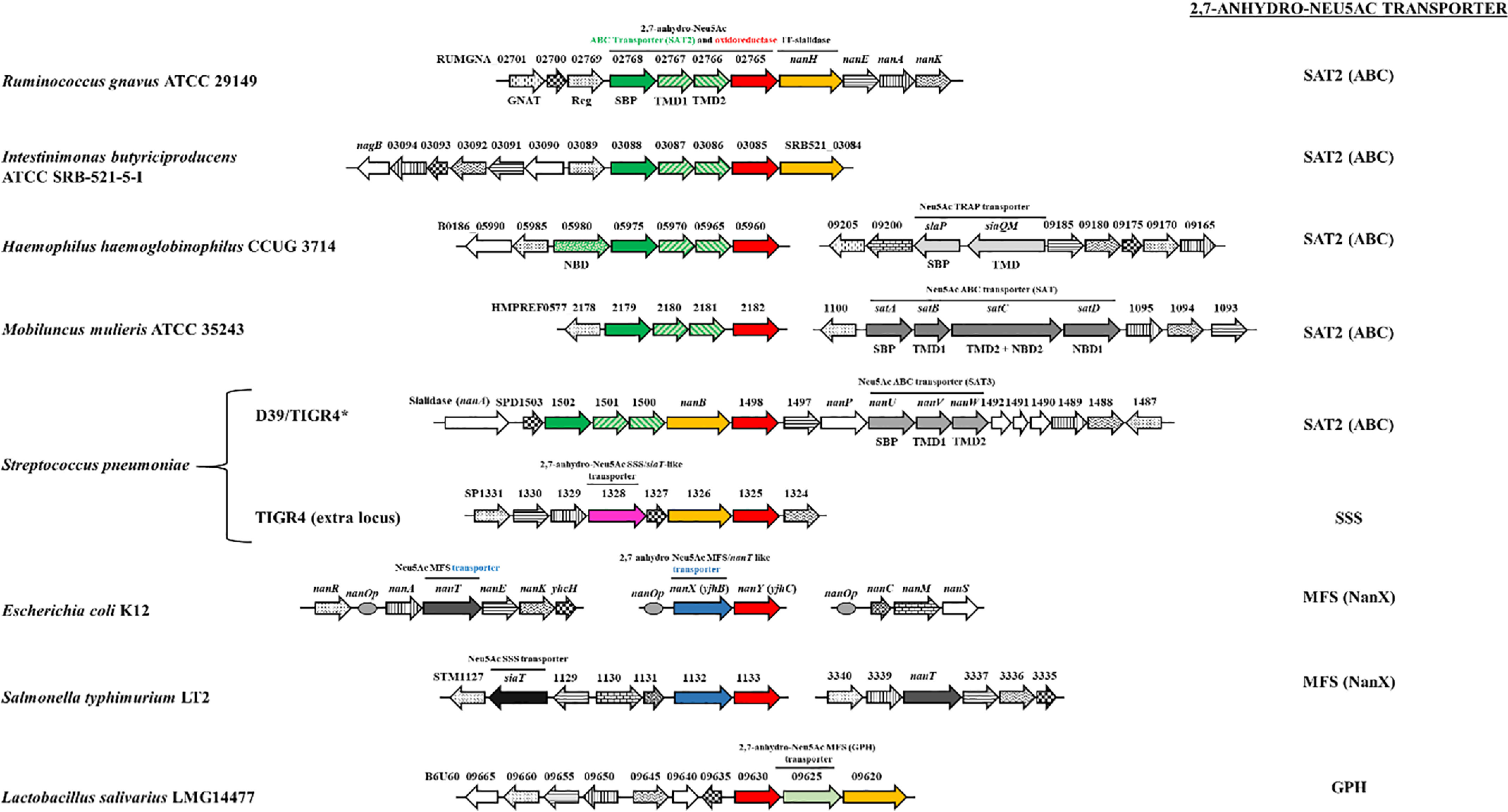
**Diversity of 2,7-anhydro-Neu5Ac catabolic clusters among sialic acid–utilizing bacteria.** Gene functions were inferred from BLAST searches followed by gene linkage and cluster analysis. Orthologous genes are identified with individual patterns. The genes encoding 2,7-anhydro-Neu5Ac transporters, 2,7-anhydro-Neu5Ac oxidoreductases, and IT-sialidases are distinguished by *color* for emphasis. Nomenclature is as follows. *nanA*, Neu5Ac lyase; *nanK*, *N*-acetylmannosamine kinase; *nanE*, *N*-acetylmannosamine-6-phosphate epimerase; *nanC*, Neu5Ac outer membrane channel; *nanM*, Neu5Ac mutarotase; *nanS*, *N*-acetyl-9-*O*-acetylneuraminate esterase; *nagB*, glucosamine-6-phosphate deaminase; *GNAT*, GCN5-related *N*-acetyltransferase; *Reg*, regulator (please note that GNAT family proteins and regulator proteins, while recurrent within clusters, may belong to different clades and thus function differently in each organism); *SAT2*, 2,7-anhydro-Neu5Ac transporter of the ABC superfamily; *siaPQM*, Neu5Ac transporter of the TRAP family; *satABCD*, Neu5Ac transporter of the ABC superfamily (SAT); *nanUVW (SAT3)*, Neu5Ac transporter of the ABC superfamily (also named *satABC*); *nanT*, Neu5Ac transporter of the MFS superfamily; *siaT*, Neu5Ac transporter of the SSS family; *nanX* (*yjhB*), 2,7-anhydro-Neu5Ac transporter (*nanT*-like) of the MFS superfamily ABC; *MFS*, major facilitator superfamily; *SSS*, sodium solute symporter family; *GPH*, glycoside-pentoside-hexuronide:cation symporter family; *SBP*, solute-binding protein; *TMD*, transmembrane domain; *NBD*, nucleotide-binding domain. Note that the classification of ABC sialic acid transporters follows Almagro–Moreno and Boyd (34). *, TIGR4 possesses both the conserved sialic acid “supercluster,” as in strain D39, and an additional, candidate 2,7-anhydro-Neu5Ac cluster bearing the *siaT*-like transporter gene. In TIGR4, the conserved supercluster bears a minor difference in the form of pseudogenes and insertions (66).

Having demonstrated that NanOx-like genes are functional in both Gram-positive and Gram-negative bacteria, functioning with different classes of transporters, we extended our analysis to likely 2,7-anhydro-Neu5Ac catabolic genes across bacterial species. Genes encoding proteins with high similarity to *Rg*NanOx (percentage identity ≥49%) were found in diverse microorganisms across the Firmicutes, Proteobacteria, and Actinobacterial phyla and were most often co-localized with other genes for sialic acid catabolism (Fig. 8). Interestingly, we showed co-occurrence of NanOx genes with known sialic acid transporters belonging to the MFS transporters, sodium solute symporter (SSS) transporters, or ABC SAT transporters. To test the hypothesis that other bacteria can act as “scavengers” of 2,7-anhydro-Neu5Ac, we heterologously expressed and purified the NanOx protein from *Hemophilus hemoglobinophilus* and showed that the recombinant protein was active against 2,7-anhydro-Neu5Ac (Fig. 6). The analysis also revealed two additional couplings of NanOx-like genes to likely 2,7-anhydro-Neu5Ac transporters, namely to transporters of the SSS family, for example in *Streptococcus pneumoniae* TIGR4 and a transporter of the GPH family in *Lactobacillus salivarius* (Fig. 8), which, together with the phylogenetically broad occurrence of the NanOx-like genes, suggests that 2,7-anhydro-Neu5Ac use is not a new trait in bacteria but the result of a symbiotic evolution of bacteria in the mammalian gastrointestinal tract.

## Discussion

Given the accessibility of sialic acids in mucus-rich environments, their utilization offers pathogenic and commensal bacteria a competitive advantage to colonize and persist within the gut (2, 19). The ability of *R. gnavus* strains to produce and metabolize 2,7-anhydro-Neu5Ac provides them with a nutritional advantage by scavenging sialic acid from host mucus in a form that they have preferential access to (4, 17–19). We previously showed that the oxidoreductase *Rg*NanOx plays a key role in the catabolism of 2,7-anhydro-Neu5Ac inside the cells by converting it into Neu5Ac, before being catabolized into GlcNAc-6-P following the canonical pathway by the successive action of NanA (Neu5Ac aldolase), NanK (ManNAc kinase), and NanE (ManNAc-6-P epimerase). However, the molecular mechanism of *Rg*NanOx enzymatic reaction remained unknown (19). Here, we showed that *Rg*NanOx acts through a multistep mechanism involving a keto intermediate and cycling of NADH/NAD^+^. The creation of a keto intermediate in sugars is widespread in biology; perhaps it is best-known for the SDR enzyme UDP-glucose/galactose epimerase (29, 30). In this enzyme, the oxidation and reduction of the sugar occur so as to invert the chirality at C4. In other enzymes, including RmlB of the dTDP-l-rhamnose biosynthetic pathway (31) and the multistep enzyme GDP-mannose 3,5-epimerase (32), the creation of a keto group and the consequent acidification of the α proton(s) allow a range of chemical reactions. In *Rg*NanOx, by creating a keto group, the enzyme has acidified the C3 proton; this facilitates an elimination reaction and formation of a conjugated intermediate 4-keto-DANA, which we detected by NMR. The NAD^+^ co-factor is tightly bound, and because in the overall reaction it is unchanged (acting as co-catalyst), there is no need to recycle it with an additional enzyme. Instead, the NAD^+^ is reduced to NADH when it creates a keto group at C4 of the substrate in the first step, and in the last step NAD^+^ is regenerated by reduction of the keto group.

Previous work using spectrophotometric assays reported that *E. coli* YjhC showed a weak interaction with Neu5Ac, with an apparent *K_m_* of 68.8 mm (20). Here, we used ESI-MS to assess the activity of YjhC against 2,7-anhydro-Neu5Ac or Neu5Ac. This analysis supported the earlier findings that YjhC could act on Neu5Ac (20) but also revealed that the enzyme was able to utilize 2,7-anhydro-Neu5Ac as a substrate in the same manner as *Rg*NanOx. Given the structural resemblance of *Rg*NanOx to YjhC, it is likely that the *E. coli* oxidoreductase also uses the same mechanism of action for the reversible conversion of 2,7-anhydro-Neu5Ac to Neu5Ac.

Further findings from our work support that the catabolism of 2,7-anhydro-Neu5Ac is not restricted to *R. gnavus* strains. *E. coli* can transport and catabolize the common sialic acid, Neu5Ac, as a sole source of carbon and nitrogen but also related sialic acids, *N*-glycolylneuraminic acid (Neu5Gc) and 3-keto-3-deoxy-d-glycero-d-galactonononic acid (KDN), which are transported via the sialic acid transporter NanT and catabolized using the sialic acid aldolase NanA (33). Here, we showed that *E. coli* BW25113 strain was able to grow on 2,7-anhydro-Neu5Ac as a sole carbon source and that the two-gene NanR-regulated operon *nanXY* (*yjhBC*) encodes both the transporter and oxidoreductase enzyme required for *E. coli* to uptake and catabolize 2,7-anhydro-Neu5Ac. This also now completes the functional characterization of all NanR-regulated genes in *E. coli* (25), giving us a broader picture of the sialic acid molecules it likely encounters in its natural environment. This ability to utilise multiple sialic acid derivatives contrasts with *R. gnavus* strains, which can only grow on 2,7-anhydro-Neu5Ac but not on Neu5Ac (19) and is consistent with *E. coli* being able to integrate diverse sialic acids into its core catabolic pathway (33). Beyond *E. coli*, our bioinformatics analyses revealed *Rg*NanOx homologues across many bacterial species that also co-occurred with predicted sialic acid transporters.

Bacteria have evolved multiple mechanisms to capture sialic acid from their environment (34, 35). To date, six different classes of sialic acid transporters have been described (35). These include the NanT MFS transporters from *E. coli* and *Bacteroides fragilis*, which in *E. coli* has been demonstrated biochemically to be a H^+^-coupled symporter (36). Secondary transporters implicated in sialic acid transport are from the SSS family present in pathogens such as *Clostridium difficile*, *Salmonella typhimurium*, or *Proteus mirabilis* (28, 37, 38). High-affinity transport of sialic acid is mediated by substrate-binding protein-dependent systems, including a tripartite ATP-independent periplasmic (TRAP) transporter, SiaPQM, and a number of different ABC transporters (39–44). The sialic acid ABC transporters have been further classified into three types: SAT, SAT2, and SAT3 (34, 35). To date, all of these transporters have been shown to transport Neu5Ac, with some being able to also transport the related sialic acids Neu5Gc and KDN (33, 37). The exception is *R. gnavus* ABC SAT2 transporter, which we demonstrated was specific for 2,7-anhydro-Neu5Ac (19). Here, we showed that in *E. coli* YjhC (NanY) is associated with a predicted sialic acid transporter, YjhB (NanX). Our genetic data suggest that YjhB could transport 2,7-anhydro-Neu5Ac but not Neu5Ac, where the previously characterized Neu5Ac transporter NanT was not able to transport 2,7-anhydro-Neu5Ac. Based on its sequence, NanX (YjhB) is classified in the MFS class of sugar transporters and is a close homologue of NanT. Our bioinformatics provide striking evidence for two additional families of secondary transporters having evolved to recognize 2,7-anhydro-Neu5Ac, namely those of the SSS and GPH families, bringing the total number of transporter families for 2,7-anhydro-Neu5Ac to four.

It is of note that *E. coli* does not encode an IT-sialidase releasing 2,7-anhydro-Neu5Ac; therefore, the ability of this strain to use 2,7-anhydro-Neu5Ac as a metabolic substrate *in vivo* would likely rely on cross-feeding in the mucosal environment. To date, only *R. gnavus* strains have been reported to produce 2,7-anhydro-Neu5Ac from Neu5Ac terminally bound glycoconjugates in the gut (4, 5, 18). Resource sharing is an important ecological feature of microbial communities living in the gut (45). Some bacteria present in the mucus might not be primary degrader but might cross-feed on mucin glycan degradation products released by other bacteria. This concept involves the ability of bacteria to benefit from substrate degradation products but also from fermentation products and plays a crucial role in microbial community shaping in the gut (46). Such cross-feeding activities have been reported in the gut mucosal environment for the utilization of Neu5Ac. For example, *Bacteroides thetaiotaomicron* VPI-5482 encodes a sialidase and can release free Neu5Ac but lacks the *nan* operon required to metabolize the liberated monosaccharide (47). On the other hand, most *C. difficile* and *S. typhimurium* subsp. enterica strains encode the *nan* operon but lack the sialidase (48) and benefit from sialidase-producing organisms such as *B. thetaiotaomicron* to acquire this nutrient from the mucosal environment (49). Our bioinformatics analyses suggest a similar diversity for 2,7-anhydro-Neu5Ac metabolism across bacterial species. For example, whereas *R. gnavus* possesses the full complement of genes to produce and utilize 2,7-anhydro-Neu5Ac, including the IT-sialidase (*Rg*NanH), the 2,7-anhydro-Neu5Ac SAT2 transporter, and the oxidoreductase (*Rg*NanOx) within the otherwise canonical Nan cluster, *E. coli* harbors a transporter with specificity for 2,7-anhydro-Neu5Ac (NanX) and the a NanOx homolog (NanY) but does not express an IT-sialidase. *Streptococcus pneumoniae* strains, on the other hand, may express up to three sialidases (neuraminidases), NanA, NanB, and NanC, of which the first two are part of a universally conserved *nan* gene cluster (42), whereas the third one is part of an additional locus present in some strains but not others (50). The conserved *nan* cluster is well-studied in strain D39 (42, 51) and is divided into three operons that include operon I (*nanA* monocistronic), operon II (the *nanB* locus), and operon III (the *nanE* locus carrying the catabolic genes) (51). The transcriptomic response of *S. pneumoniae* D39 to Neu5Ac clearly demonstrated that NanR acts as a transcriptional activator of the *nan* operons I and III in the presence of Neu5Ac, but not of operon II, for which regulation mechanisms remained unknown (51). Because NanB has been functionally characterized as an IT-sialidase in *S. pneumoniae* (52) and the *nan* operon II also contains a gene encoding an oxidoreductase and a SAT2 ABC transporter (as in the case of *R. gnavus*), our results strongly suggest that the *nan* operon II is dedicated to 2,7-anhydro-Neu5Ac utilization. This is also in agreement with the reported growth assays of *S. pneumoniae* transporter mutants, showing that SAT3 was required for Neu5Ac transport but that growth on Neu5Ac was unaffected in the SAT2 mutant (42), suggesting that SAT2 may be involved in 2,7-anhydro-Neu5Ac, although this remains to be tested experimentally. The existence of multiple transporters with different specificities for sialic acid derivatives within the same species (*e.g. E. coli* NanT/YjhB) or restricted to 2,7-anhydro-Neu5Ac (*e.g. R. gnavus* SAT2) points toward divergent evolution of a common ancestor. Together, these data demonstrate that 2,7-anhydro-Neu5Ac catabolism is not exclusive to *R. gnavus* and may help shape microbial communities in the gut. From an ecological point of view, because *R. gnavus* is the only strain reported to produce 2,7-anhydro-Neu5Ac in the gut, the strict specificity of its sialic acid transporter may give it a nutritional advantage while maintaining its keystone status in the mucus niche by providing an important nutrient to the microbial community.

## Experimental procedures

### Materials and strains

All chemicals were obtained from Sigma unless otherwise stated. The auto-induction medium “Terrific Broth Base with Trace Elements” was purchased from ForMedium (Dundee, UK). 2,7-Anhydro-Neu5Ac was produced as reported by Bell *et al.* (19). The *E. coli* Δ*nanT*, Δ*yjhB* (JW5768), and Δ*yjhC* (JW5769) mutants all come from the KEIO collection (53) and are derivatives of BW25113, which we used as WT strain. The Δ*nanT* strain, which we have characterized previously (28, 36) carries an unmarked deletion, whereas the Δ*yjhB* and Δ*yjhC* mutants are unmodified and retain the original Kan marker.

### Bacterial growth assays

Growth curves of *E. coli* BW25113 and sialometabolism mutants (Δ*nanT*, Δ*yjhC*, and Δ*yjhB*) before or after complementation were carried out in M9 medium (without glucose) supplemented with 11.1 mm Neu5Ac, 2,7-anhydro-Neu5Ac, or glucose using 200-µl cultures in 96-well microtiter plates. The OD_595 nm_ was measured every 30 or 60 min for 16 h in a FLUOstar OPTIMA (BMG LABTECH).

### Complementation of E. coli ΔyjhC

Plasmid pES156 is a derivative of the low-copy plasmid pWKS30 (67) carrying *E. coli yjhBC* under the control of the *lac* promoter. To make pES156, a PCR product for the *yjhBC* genes was amplified with primers E549 and E550 (Table S2) cut with Eco31I (the primers carried sites for this type IIS enzyme that were designed to produce Acc65I- and BamHI-compatible ends) and ligated into pWKS30. The constructs were verified by sequencing. The resulting plasmid was transformed into chemically competent Δ*yjhC* cells using heat shock. For bacterial growth curves, 1 mm isopropyl 1-thio-β-d-galactopyranoside was added to the growth medium for induction of the *yjhBC* proteins.

### Cloning, site-directed mutagenesis, heterologous expression, and protein purification

*Rg*NanOx was heterologously expressed and purified as described previously (19). Mutants of *Rg*NanOx were generated using the NZYMutagenesis kit (NZYTech) using the primers listed in Table S2 following the manufacturer's instructions, expressed, and purified as described previously for the WT enzyme (19). Fractions collected following gel filtration were analyzed using NuPAGE Novex 4–12% BisTris gels (Life Technologies). Fractions were pooled and concentrated using a 10,000 molecular weight cut-off Vivaspin column (Vivaspin, Germany). Protein concentration was determined by a NanoDrop spectrophotometer (Thermo Scientific) using extinction coefficients calculated by Protparam (ExPASy-Artimo, 2012) from the peptide sequence.

The oxidoreductase genes encoding YjhC from *E. coli* BW25113 and the homologue in *H. hemoglobinophilus* CCUG 3714 (B0186_5960; strain purchased from DSMZ, reference no. DSM21241) were amplified from genomic DNA with HerculaseIIFusion (Agilent) using primer pairs E521 plus E522 and ES525 plus ES526, respectively (Table S2).The PCR products, each flanked by sites for type IIS enzyme Eco31I (BsaI) designed to produce NcoI and XhoI compatible ends, were digested with Eco31I and ligated in pET28a digested with NcoI and XhoI. The resulting constructs were confirmed by sequencing.

For purification of these recombinant proteins, the corresponding expression plasmids were transformed into BL21(DE3) pLysS, and single colonies were grown overnight in 10 ml of lysogeny broth with Cm15 Kan25. These starter cultures were used to inoculate 1 liter of Terrific Broth (Terrific Broth 47.6 g/liter, glycerol 0.5% (v/v)) with antibiotics. Cultures were grown in baffled flasks at 37 °C and 200 rpm until 0.7 OD_650_ before being quickly chilled on ice-water for about 20 min and finally induced overnight with 0.2 mm isopropyl 1-thio-β-d-galactopyranoside at 18 °C and 220 rpm. After harvest, pellets were resuspended in equilibration buffer (50 mm KP_i_, 200 mm NaCl, 20% glycerol, 40 mm imidazole, pH 7.8) and disrupted with sonication. The clarified lysate was run through an immobilized metal affinity chromatography column to elute the C-terminal His_6_-tagged proteins, which eluted in sharp peaks with a single 500 mm imidazole step. These were further purified by size-exclusion chromatography with elution in 50 mm KP_i_, 200 mm NaCl, pH 7.8, dialyzed against assay buffer (20 mm Tris, 150 mm NaCl, 2 mm tris(2-carboxyethyl)phosphine, pH 7.5), and finally concentrated to 0.5–1 mm for storage at 4 °C. Purity was assessed on SDS-PAGE as above.

### ESI-MS analysis of the oxidoreductase reaction

To assay for oxidoreductase activity, the purified recombinant proteins were incubated in 100-μl reactions at 37 °C overnight with 1 mg/ml 2,7-anhydro-Neu5Ac or Neu5Ac in 20 mm sodium phosphate buffer, pH 7.5, in the presence 500 μm NADH. The conversion of 2,7-anhydro-Neu5Ac to Neu5Ac or Neu5Ac to 2,7-anhydro-Neu5Ac was monitored by ESI-MS. Briefly, 100 µl of acetonitrile was added to each reaction, vortexed, and centrifuged to remove particles. The resulting supernatants were loaded onto an AmaZon Speed ETD (Bruker) mass spectrometer and analyzed by direct injection in negative mode.

### NMR analyses of the oxidoreductase reaction

All 1D NMR experiments were performed using a Bruker Advance I 500-MHz spectrometer with a 5-mm PATXI ^1^H/D-^13^C/^15^N Z-GRD probe at 293 K. To follow the kinetics of the reaction and assess the position of deuteration, two samples containing 2 mm 2,7-anhydro-Neu5Ac, 100 μm NADH, and 60 μm *Rg*NanOx were used, one in deuterated PBS buffer (PBS/D_2_O) and one in standard PBS buffer (PBS/H_2_O, containing 10% D_2_O for locking purposes). The reaction was followed by acquiring 1D NMR experiments at 15-min intervals over 24 h. The standard *zg* pulse sequence was used for the D_2_O sample, whereas excitation sculpting was used to remove the strong solvent signals for the H_2_O sample (pulse sequence: *zgesgp*). 1 mm DSS-*d*_6_ was added to each sample as an internal reference, and the signal was calibrated to 0 ppm. All 2D NMR experiments were performed using an Avance NEO 600-MHz NMR spectrometer equipped with He-cooled TCI cryoprobe. Typically, a spectral width of 12 ppm was used for ^1^H and 165 ppm for ^13^C, acquiring 8 scans/experiment with a time domain of 2048 data points in the direct dimension and 128 experiments in the indirect dimension. To characterize the reaction intermediate, a sample containing 3 mm 2,7-anhydro-Neu5Ac, 100 μm NADH, 15 μm *Rg*NanOx, and 1 mm DSS-*d*_6_ in PBS/D_2_O was prepared and analyzed at 293 K. A full set of 2D NMR experiments, including HSQC (*hsqcetgpsi*), COSY (*cosygpqf*), TOCSY (*mlevph*) and TOCSY-HSQC (*hsqcdietgpsi*), was acquired to fully assign ^1^H and ^13^C signals of the intermediate.

### DSF

The Applied Biosystems StepOnePlus Real-Time PCR system (Life Technologies) was used to record the thermal stability of the purified proteins with and without substrates or cofactors. Reactions were performed in 20 mm sodium phosphate, pH 7.5, and consisted of 5 μm protein, 5× SYPRO Orange (prepared as a 40× stock), 10 mm substrate (2,7-anhydro-Neu5Ac or Neu5Ac), 1 mm cofactor (NAD or NADH) in a 20-μl final reaction volume. Samples were heated from 10 to 99 °C in 0.5 °C increments, taking fluorescent readings at each time point. Triplicate measurements were performed for each sample. Negative controls were included for each component of the experiment individually as well as a dye-only control well. The melting point (*T_m_*) of each sample in °C was obtained from the lowest point of the first derivative plot.

### Crystallization, data collection, and structure determination of RgNanOX

Sitting-drop vapor diffusion crystallization experiments of *Rg*NanOx were set up at a concentration of 20 mg/ml. The structure was acquired from a crystal grown in the JCSG Plus screen (100 mm sodium citrate, pH 5.5, 20% PEG 3000). The diffraction experiment was performed on the I04 beamline at Diamond Light Source Ltd. at 100 K using a wavelength of 0.9795 Å. The data were processed with Xia2 pipeline. The structure was phased using online MrBUMP Sculptor pipeline (54) with PDB entry 5UI9. Refinement was carried out using Phenix AutoBuild (55), Refmac (56), and PDB REDO (57). Coot (58) was used for manual model building and Molprobity (59) for structure validation. It has not yet been possible to obtain well-diffracting crystals of any substrate analog complex of the protein. For modeling, after manual positioning in COOT, the structure was idealized using REFMAC5 (56).

### Bioinformatics

To search and compare protein sequences for *Rg*NanOx, the BLAST program and BLASTp (60) were used. The DALI server (61) was used for protein structure comparison. Amino acid sequences and atomic structures of homologues were sourced from the NCBI/UniProt and PDB databases, respectively. BLAST searches were initiated with *Rg*NanOx/YjhC as queries followed by manual annotation of the ORFs going outward. To map complete sialometabolic pathways within individual microorganisms, BLAST searches were performed against all known Neu5Ac transporters (35) as well as for the Neu5Ac aldolase NanA and *N*-acetylmannosamine-6-phosphate epimerase NanE (using queries of different organismal origin). This was carried out to include annotation of genes for *Rg*NanOx homologues residing outside the cluster or where multiple sialometabolic clusters were present.

## Data availability

The atomic coordinates have been deposited in the Protein Data Bank under accession codes 6Z3B (2.58 Å resolution structure of *R*gNanOx), and 6Z3C (1.74 Å resolution structure of *R*gNanOx). All other data supporting the findings of this study are available within the article and the supplemental information.

## Supplementary Material

Supporting Information
