# Supplementary material for: Uncovering a novel molecular mechanism for scavenging sialic acids in bacteria
Source: J Biol Chem. 2020 Jul 15;295(40):13724–36. doi: 10.1074/jbc.RA120.014454 (PMC7535918; doi:10.1074/jbc.RA120.014454)
Supplement: Supporting Information [file supp_295_40_13724__index.html]

Uncovering a novel molecular mechanism for scavenging sialic acids in bacteria — Structural basis of 2,7-anhydro-Neu5Ac catabolism — Uncovering a novel molecular mechanism for scavenging sialic acids in bacteria — Structural basis of 2,7-anhydro-Neu5Ac catabolism — Supporting Information 

# Uncovering a novel molecular mechanism for scavenging sialic acids in bacteria

## Supporting Information

- Supporting Information (to be published online) - Supplemental file
