# Supplementary material for: Uncovering a novel molecular mechanism for scavenging sialic acids in bacteria
Source: J Biol Chem. 2020 Jul 15;295(40):13724–36. doi: 10.1074/jbc.RA120.014454 (PMC7535918; doi:10.1074/jbc.RA120.014454)
Supplement: Supporting Information [file supp_RA120.014454_160975_2_supp_563611_qdhxg7.docx]

**Supplementary information**

**Supplementary Figures**


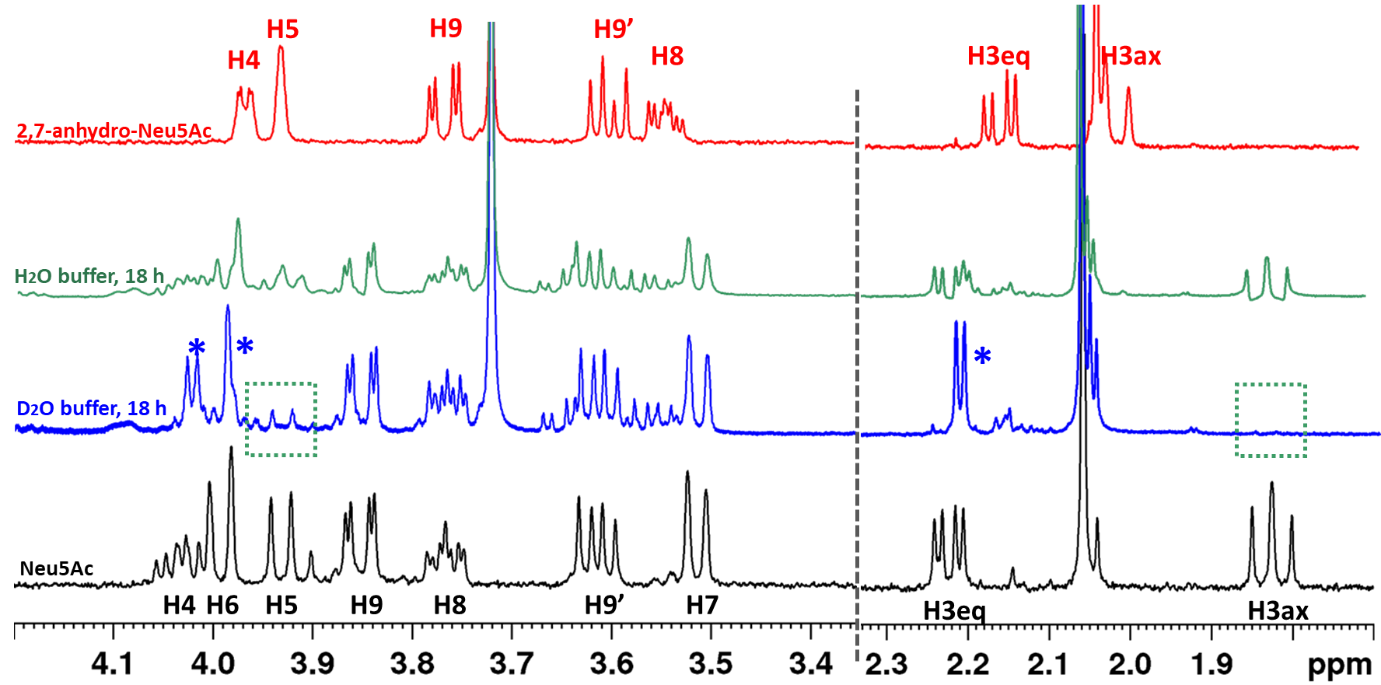


**Supp. Fig. 1:** Comparison of the 1D spectra of Neu5Ac (black), 2,7-anhydro-Neu5Ac (red), and final time points of the reaction carried out H_2_O buffer (green) and D_2_O buffer (blue). In the final time point of the reaction carried out in D_2_O buffer (blue), the dotted squares highlight the absence of H5 and H3ax; while the blue stars highlight the neighbouring signals, which multiplicity became simplified, as a results of the protons absence.


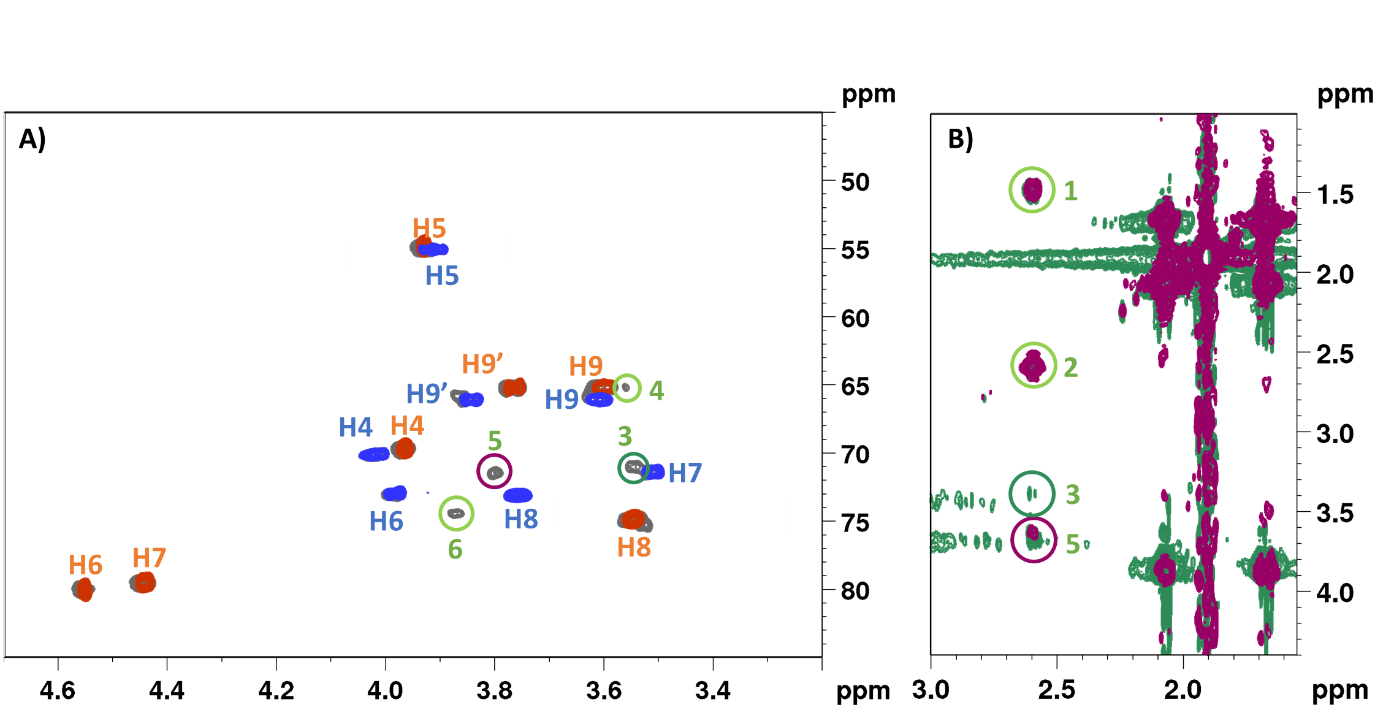


**Supp. Fig. 2:** Homo-nuclear 2D NMR analysis of the enzymatic reaction mixture containing the keto-sugar intermediate. ^1^H,^1^H COSY (purple) and ^1^H,^1^H TOCSY (dark green) spectra of the reaction mixture. The COSY and TOCSY correlations allow us to assign protons H6 and H7 of the 4-keto-DANA intermediate. Based on these data, the unknown signals (see Figs. 1B and 1C in the main text) were assigned as: signal 1 = amidic CH_3_, signal 2 = H5, signal 3 = H7, signal 5 = H6 of the 4-keto-DANA intermediate.


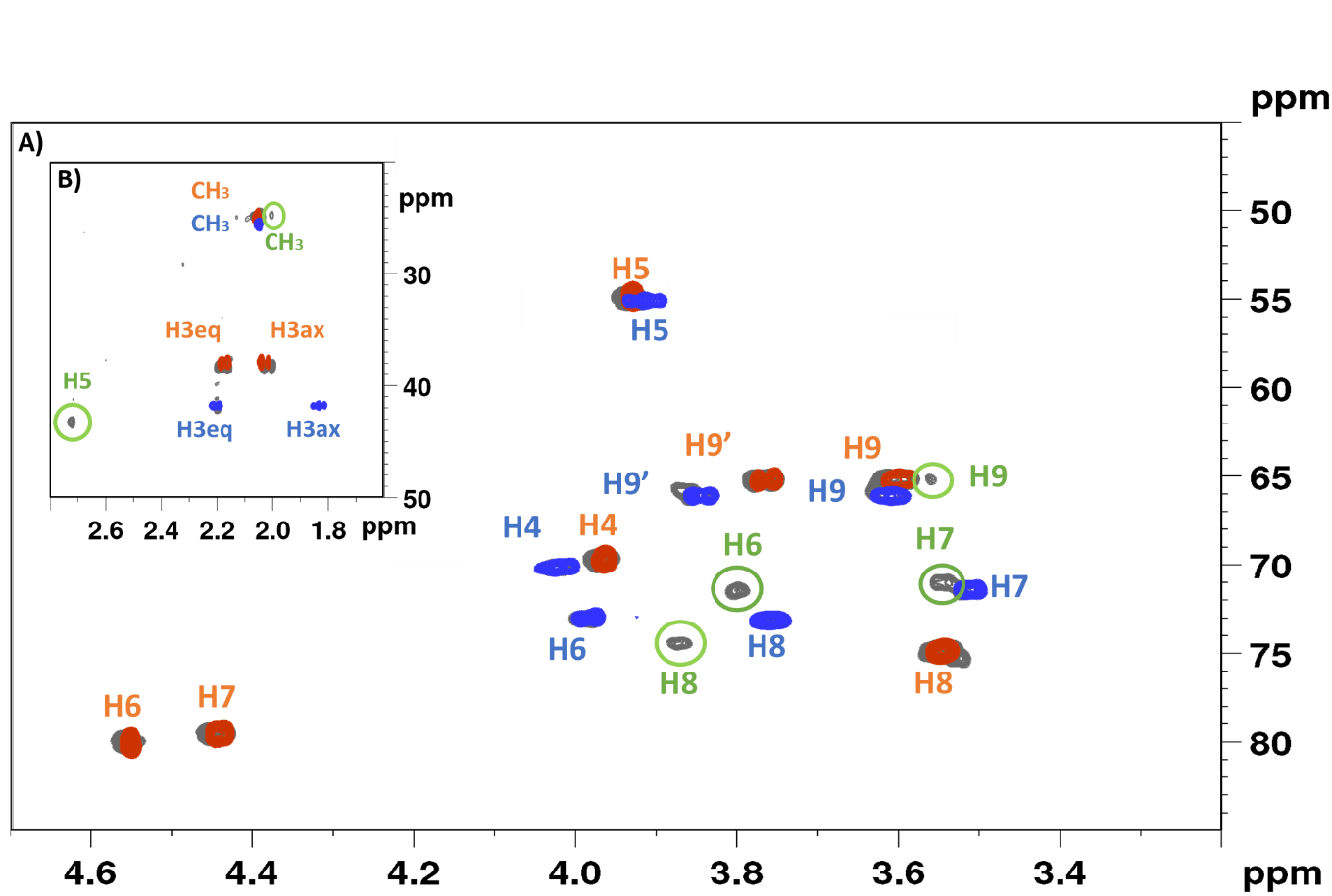


**Supp. Fig. 3:** Full assignment of the 4-keto-DANA intermediate. To ease the interpretation, Figs. 1B and 1C of the main text are intentionally repeated here including the intermediate cross peaks full assignments (green circles and labels). **A)** Zoom on the typical sugar-ring spectral region. **B)** Zoom on the aliphatic/α protons spectral region.

**Supplementary Tables**

**Supp. Table 1:**. 4-keto-DANA ^1^H and ^13^C assignment.

| Signal | ^1^H chemical Shift (ppm) | ^13^C chemical Shift (ppm) | Assignment |
| --- | --- | --- | --- |
| 1 | 2.00 | 24.7 | CH_3_ |
| 2 | 2.72 | 43.1 | H5 |
| 3 | 3.55 | 70.9 | H7 |
| 4 | 3.56 | 65.1 | H9 |
| 5 | 3.80 | 71.4 | H6 |
| 6 | 3.87 | 74.4 | H8 |

**Supp. Table 2:** Primers used in this study

| *Rg*NanOx K93A F | GTGAATGTATTTTGTGAAGCACCCATTGCACTTTCCTATCAG | *Rg*NanOx SDM primers |
| --- | --- | --- |
| *Rg*NanOx K93A R | CTGATAGGAAAGTGCAATGGGTGCTTCACAAAATACATTCAC |  |
| *Rg*NanOx K163A F | ACAGCAGCCAACTATTTCCTGGGCAAAAATCAGGGAAAAAT |  |
| *Rg*NanOx K163A R | ATTTTTCCCTGATTTTTGCCCAGGAAATAGTTGGCTGCTGT |  |
| *Rg*NanOx H175A F | GGTGGACATCTGTACGCTCACATCCATGAATTGGATTGTGTAC |  |
| *Rg*NanOx H175A R | GTACACAATCCAATTCATGGATGTGAGCGTACAGATGTCCACC |  |
| *Rg*NanOx H176A F | GGTGGACATCTGTACCATGCCATCCATGAATTGGATTGTGTAC |  |
| *Rg*NanOx H176A R | GTACACAATCCAATTCATGGATGGCATGGTACAGATGTCCACC |  |
| E549 | GGAGCTGGTCTCGGTACCGTAAAAGAAGGAGATATACATATGGCAACAGCATGGTATAAAC | pES156 construction |
| E550 | CACTGCGGTCTCGGATCCTTACATTACTGATGTATGTTTAATGATC |  |
| E521 | GACGATGGTCTCACATGATTAATTATGGCGTTGTTGGTG | *Ec*YjhC |
| E522 | CTCGTCGGTCTCCTCGAGCATTACTGATGTATGTTTAATGATC |  |
| E525 | GACGATGGTCTCACATGATGAAATATGGTGTTGTTGGCG | *Hh*YjhC |
| E526 | CTCGTCGGTCTCCTCGAGGCCAGTAATCTCCGTTAAGTTCAC |  |
